# Correction: Radiolabeling and *in vivo* evaluation of lanmodulin with biomedically relevant lanthanide isotopes

**DOI:** 10.1039/d3cb90017g

**Published:** 2023-07-10

**Authors:** Kirsten E. Martin, Joseph A. Mattocks, Dariusz Śmiłowicz, Eduardo Aluicio-Sarduy, Jennifer N. Whetter, Jonathan W. Engle, Joseph A. Cotruvo, Eszter Boros

**Affiliations:** a Department of Chemistry, Stony Brook University Stony Brook New York 11794 USA eszter.boros@stonybrook.edu; b Department of Chemistry, The Pennsylvania State University University Park Pennsylvania 16802 USA juc96@psu.edu; c Department of Medical Physics, University of Wisconsin Madison Wisconsin 53705 USA; d Department of Radiology, University of Wisconsin Madison Wisconsin 53705 USA

## Abstract

Correction for ‘Radiolabeling and *in vivo* evaluation of lanmodulin with biomedically relevant lanthanide isotopes’ by Kirsten E. Martin *et al.*, *RSC Chem. Biol.*, 2023, https://doi.org/10.1039/D3CB00020F.

The authors regret the omission of a funding body associated with the work. J. A. M. and J. A. C. acknowledge funding from the US Department of Energy (DE-SC-0021007 to J. A. C.).

The Royal Society of Chemistry apologises for these errors and any consequent inconvenience to authors and readers.

## Supplementary Material

